# A multi-component psychosocial intervention programme to reduce psychological distress and enhance social support for women undergoing termination of pregnancy for foetal anomaly in China: A randomised controlled trial

**DOI:** 10.1016/j.ijnsa.2025.100389

**Published:** 2025-07-29

**Authors:** Chunxiang Qin, Ying Li, Ying Wang, Chi Huang, Gui Xiao, Lihong Zeng, Yanjuan He, Wei Jiang, Jiaying Xie

**Affiliations:** a Department of Nursing, the Third Xiangya Hospital, Central South University, Changsha, China; b Health Management Medicine Center, the Third Xiangya Hospital, Central South University, Changsha, China; c Department of Pediatrics, the Third Xiangya Hospital, Central South University, Changsha, China; d Xiangya School of Nursing, Central South University, Changsha, China; e Department of Nursing, Jiangxi University of Traditional Chinese Medicine, Nanchang, Jiangxi, China; f Department of Obstetrics, Changsha Hospital for Maternal and Child Health Care, Changsha, China; g Department of Gynaecology, Hunan Provincial Maternal and Child Health Care Hospital, Changsha, China; h School of Nursing and Midwifery, University of Galway, Galway, Republic of Ireland

**Keywords:** Termination of pregnancy for foetal anomaly, Psychosocial intervention, Depression, Posttraumatic stress, Psychological flexibility, Acceptance and Commitment Therapy

## Abstract

**Background:**

Termination of pregnancy for foetal anomaly causes significant psychological distress, yet evidence-based psychosocial interventions tailored to the needs of women experiencing termination of pregnancy for foetal anomalyremain limited.

**Objective:**

To evaluate the effectiveness of a multi-component psychosocial intervention designed to reduce depression and post-traumatic stress disorder (PTSD) and enhance psychological flexibility and social support among women following termination of pregnancy for foetal anomaly.

**Methods:**

A single-blinded, two-arm randomised controlled trial was conducted in two maternity hospitals in Hunan Province, China. Eighty-six participants were randomly allocated to the multi-component psychosocial intervention group (*n* = 41) or the control group (*n* = 45). The multi-component psychosocial intervention included informational support, Acceptance and Commitment Therapy, and social support involving an online peer support group and family engagement. Depression, PTSD, psychological flexibility and social support were assessed at baseline, immediately (T1), one-month (T2) and three-months (T3) post-intervention.

**Results:**

Although the intervention group showed greater reductions in depressive symptoms (EPDS: *β* = 0.92, 95 % CI: –1.38 to 3.21, *p* = 0.435) and post-traumatic stress symptoms (IES-R: *β* = 5.31, 95 % CI: –1.25 to 11.86, *p* = 0.113) compared to the control group, these differences did not reach statistical significance. Significant group-by-time effects emerged for PTSD-related avoidance symptoms (*β* = 2.98, 95 % CI: 0.27 to 5.70, *p* = 0.031; *d* = 0.49), perceived social support (*β* = –1.56, 95 % CI: –3.10 to –0.02, *p* = 0.047; *d* = 0.38) and utilisation of social support (-0.83, 95 % CI: -1.48 to -0.18, *p* = 0.013; *d* = 0.55) at T3. Participants with baseline EPDS > 9 (*n* = 54) showed stronger effects, with significant improvements in depression (*β* = 2.02, 95 % CI: 0.38 to 3.66, *p* = 0.016) and experiential avoidance (*β* = 2.54, 95 % CI: 0.30 to 4.78; *p* = 0.026) at T1, PTSD (*β* = 11.75, 95 % CI: 2.39 to 21.12, *p* = 0.014; *d* = 0.61) and utilisation of social support (*β* = -0.95, 95 % CI: -1.85 to -0.04; *p* = 0.040, *d* = 0.65) at T3. No adverse events occurred.

**Conclusions:**

The multi-component psychosocial intervention programme reduced PTSD-related avoidance symptoms and enhanced social support. Participants with depressive symptoms experienced immediate improvements in depression and psychological flexibility, with sustained benefits in PTSD and utilisation of social support over three months. Tailoring the intervention components to individual needs may benefit women undergoing termination of pregnancy for foetal anomaly. Further research should compare women with and without baseline psychological distress to determine who benefits most from this intervention.

**Trial Registration:**

Chinese Clinical Trial Registry: ChiCTR2100047195.

**Contribution of the paper**

**What is already known about the topic**

• Termination of pregnancy for foetal anomaly leads to significant psychological distress.

• The needs of women undergoing termination of pregnancy for foetal anomaly are complex and evolve.

• Evidence-based psychosocial interventions tailored to these changing needs remain limited

**What this paper adds**

• A multi-component psychosocial intervention programme effectively reduced PTSD-related avoidance symptoms and enhanced social support for women undergoing termination of pregnancy for foetal anomaly.

• Women with depressive symptoms showed significant improvements in depression and psychological flexibility immediately after the intervention, with sustained benefits in post-traumatic stress symptoms and utilisation of social support for up to three months.

• While this intervention addresses the complex needs of women following termination of pregnancy for foetal anomaly, its implementation should be flexible and tailored to individual psychological conditions and support needs.

Alt-text: Unlabelled box

## 1 Background

Psychological distress is highly prevalent among women undergoing termination of pregnancy for foetal abnormalities, a burden that is particularly pronounced in low- and middle-income countries, where the prevalence of foetal abnormalities is relatively high. Globally, foetal abnormalities are detected in approximately 2 % to 3 % of pregnancies annually (Dolk et al., 2010). In China, the prevalence is estimated at 5.6 %, translating to approximately 900,000 new cases each year (2012). Among these cases, an estimated 90.7 %, or roughly 820,000 pregnancies, result in termination following prenatal diagnosis (Xie et al., 2020).

Beyond the medical and ethical complexities, termination of pregnancy for foetal anomaly has profound psychological consequences for expectant mothers, significantly increasing the risk of mental health disorders such as depression and post-traumatic stress disorder. Kersting et al. (2009) reported that 22.4 % of women who underwent termination of pregnancy for foetal anomaly were diagnosed with a psychiatric disorder, including post-traumatic stress disorder, depression, or anxiety, which is almost four times the rate observed in women following normal childbirth. A longitudinal study further demonstrated that nearly half of the women who underwent termination of pregnancy for foetal anomaly continued to experience pathological levels of post-traumatic stress symptoms and depression 16 months after the termination, compared to 4 months (Korenromp et al., 2009). Moreover, this psychological distress can persist, negatively impacting subsequent pregnancies (Hunter et al., 2017) and contributing to the economic burden associated with perinatal mental illness (Bauer et al., 2016).

Two modifiable factors, psychological flexibility and social support, have emerged as critical buffers against psychological distress in women experiencing termination of pregnancy for foetal anomaly. Psychological flexibility, defined as the capacity to remain present and engage in values-based action despite distressing experiences, has been associated with reduced depressive symptoms in the context of traumatic life events (Fonseca et al., 2020). Research suggests that higher levels of psychological flexibility are linked to better coping and lower depression risk among women following termination of pregnancy for foetal anomaly (Hanschmidt et al., 2018; Li et al., 2022). Similarly, social support—including objective, perceived, and utilised support from partners, family, and peers—plays a key role in alleviating negative emotional states. Several studies have confirmed that adequate social support is associated with lower levels of post-traumatic stress and depression in this population (Barros et al., 2013; Chen et al., 2024; Li et al., 2017).

Psychosocial interventions have been described as a group of nonpharmacological therapeutic interventions grounded in psychological theory, which aim to address the psychological, social, personal and relational problems of people with mental health conditions (Barbui et al., 2020; Xie et al., 2024). Previous studies indicate that psychosocial interventions, such as counselling, cognitive-behavioural therapy, expressive writing, family support, and peer support, can reduce psychological distress and improve emotional well-being in women undergoing termination of pregnancy for foetal anomaly (Xie et al., 2022). However, the complexity and variability of psychosocial needs following termination of pregnancy for foetal anomaly suggest that a combination of tailored intervention components may be required for optimal effectiveness (Shaohua and Shorey, 2021; Zhou et al., 2023).

To address the complex needs of women undergoing termination of pregnancy for foetal anomaly, a multi-component psychosocial intervention programme was developed by Wang et al. (2024). This programme is informed by the expressed needs (Zhou et al., 2023) and guided by the cognitive-emotional-behavioural framework of women undergoing termination of pregnancy for foetal anomaly (Qin et al., 2019). This multi-component psychosocial intervention programme is designed to provide integrated informational, psychological, emotional and social support for women undergoing termination of pregnancy for foetal anomaly. However, its effectiveness on psychological well-being for women undergoing termination of pregnancy for foetal anomaly remains unknown. Thus, this study reports the findings of a randomised controlled trial evaluating the effectiveness of the multi-component psychosocial intervention in improving depression, post-traumatic stress symptoms, psychological flexibility, and social support among women following termination of pregnancy for foetal anomaly.

## 2 Methods

### 2.1 Study design

This study employed a single-blinded, two-arm, parallel-group randomised controlled trial with a repeated-measures design to evaluate the effects of the multi-component psychosocial intervention programme amongst women undergoing termination of pregnancy for foetal anomaly. The study was conducted and reported in accordance with the Consolidated Standards of Reporting Trials Statement for Social and Psychological Interventions (CONSORT SPI 2018) (Grant et al., 2018). This study was prospectively registered in the Chinese Clinical Trial Registry (Registration Number: ChiCTR2100047195).

### 2.2 Participants

Recruitment occurred between August 2021 and January 2022. Participants were recruited from the obstetrics and gynaecology wards of the two largest maternity service institutions in Hunan Province, China, which had comparable care protocols and patient characteristics. Eligible participants were women aged 18 years or older (Rocha et al., 2018), diagnosed with foetal anomaly, who were undergoing termination of pregnancy, capable of using smartphones, and able to provide informed consent. Initially, eligibility was limited to those with scores of the Edinburgh Postnatal Depression Scale >9. After a pilot study, the criteria were broadened to include all women undergoing termination of pregnancy for foetal anomaly due to the limited population size. No participants were recruited for the main trial prior to this change. Exclusion criteria included continuation of pregnancy, severe life-threatening complications (e.g., heart failure, eclampsia), pre-existing diagnosed mental disorders, or engagement in psychotherapy within the past 30 days. Withdrawal criteria included participant-requested withdrawal or inability to continue due to health issues.

### 2.3 Sample size

The sample size was calculated using the Power Analysis & Sample Size System (PASS) version 11. Primary outcome parameters were informed by previous studies (Qian et al., 2021; Sun et al., 2018), with values for the Edinburgh Postnatal Depression Scale (EPDS, *δ* = 6.00, *s* = 4.96) and the Impact of Event Scale-Revised (IES-R, *δ* = 4.12, *s* = 7.24). Assuming a power of 0.90, a two-tailed alpha of 0.05, and a 20 % attrition rate, a total of 43 participants per group were required.

### 2.4 Randomisation and blinding

A combination of simple random sequences and unequal block sizes with a 1:1 allocation ratio was used to ensure allocation unpredictability and balance (Schulz and Grimes, 2018). The researcher, A, used an online random number generator (https://www.random.org/) to generate the random allocation sequence. The mixed randomisation scheme used in this study is described in detail in Supplementary Material 1. Researcher B enrolled the participants. Allocation was concealed and administered by researcher A via WeChat (a widely used mobile communication app in China), maintaining separation from recruitment and allocation. The outcome assessor (researcher C) was blinded to group allocation, while the nature of the intervention precluded blinding of the intervention provider (researcher B) and participants.

### 2.5 Intervention

The intervention used in this study, the multi-component psychosocial intervention programme, was developed based on the needs and the cognitive-emotional-behavioural framework of women undergoing termination of pregnancy for fetal anomaly. The development of this programme, including its theoretical framework and intervention structure, has been previously published (Qin et al., 2019; Wang et al., 2024; Zhou et al., 2023).

### 2.5.1 Intervention group

Participants in the intervention group received routine hospital care alongside the multi-component psychosocial intervention programme, which aims to support women after admission. The multi-component psychosocial intervention programme consists of three integrated components:

(1) informational support, delivered through printed leaflets and digital messages, aimed to enhance participants’ understanding of and preparedness for termination procedures and related experiences.
(2) emotional and psychological support, based on Acceptance and Commitment Therapy principles, provided through individual face-to-face sessions conducted by trained facilitators; and
(3) social support, offered through structured peer support group interactions on WeChat and educational guidance for family involvement.

Peer support facilitators were invited from a previous study (Huang, 2021) and trained according to the peer support programme developed by Jieqiong et al. (2018). Each participant received four face-to-face sessions based on Acceptance and Commitment Therapy principles, each lasting 40–60 min, delivered at the bedside in curtained-off areas or private rooms when available. These sessions were facilitated by researcher B, a nurse who had completed Acceptance and Commitment Therapy training and obtained certification in the field. Detailed intervention content and delivery method outlines are provided in Supplementary Material 2. To enhance accessibility and promote sustained engagement, digital components were delivered through an intervention platform based on WeChat, as presented in Supplementary Material 3.

#### 2.5.2 Control group

Participants in the control group received the standard care, which did not routinely include any psychological interventions. No extra interventions were provided by researchers.

### 2.6 Measurements

Eligibility screening was performed by nurses. Eligible participants who provided informed consent were randomly allocated to either the intervention or control group following baseline evaluation. Questionnaires were completed via WeChat at baseline (T0), immediately after the intervention (T1), one month after the intervention (T2), and three months after the intervention (T3).

Demographic and clinical data were collected at baseline using a self-designed questionnaire, which included age, residence, education, employment, income, insurance status, religion, gestational history, number of existing children, gestational weeks, and recent negative life events.

#### 2.6.1 Primary outcomes

The primary outcomes were the group by time effects in psychological distress, specifically depressive and post-traumatic stress symptoms at T3. Depressive symptoms were assessed using the Chinese version of the Edinburgh Postnatal Depression Scale (Cox et al., 1984; Lee et al., 1998). Cronbach’s *α* was 0.87 (Cox et al., 1984). The scores of the Edinburgh Postnatal Depression Scale >9 indicate mild depression, and scores >12 indicate at least moderate depression (Agostini et al., 2019; Shrestha et al., 2016; Stasik-O'Brien et al., 2019). Posttraumatic stress symptoms were assessed using the Impact of Event Scale-Revised, covering intrusion, avoidance, and hyperarousal symptoms. Scores >19 suggest clinical concern, and >33 indicate high post-traumatic stress disorder risk (Wilson and Tang, 2007). Higher scores denote more severe symptoms (Hosey et al., 2019; Park et al., 2021). The Chinese version demonstrated excellent reliability, with a Cronbach's α of 0.96 (Huang et al., 2006).

#### 2.6.2 Secondary outcomes

The secondary outcomes included changes in psychological flexibility and social support level. Psychological flexibility was assessed using the Acceptance and Action Questionnaire-II (Paladines-Costa et al., 2021; Rochefort et al., 2018) and the Cognitive Fusion Questionnaire-Fusion (Barney et al., 2021; Lucena-Santos et al., 2017; Zhang et al., 2014). The Acceptance and Action Questionnaire-II is a 7-item Likert scale (score range: 7–49), with higher scores indicating greater experiential avoidance. The Cognitive Fusion Questionnaire-Fusion is a 9-item Likert scale (score range: 9–36), with higher scores indicating greater cognitive fusion. The psychological flexibility score was derived by summing the total scores of both measures. Higher scores indicate greater psychological inflexibility and lower psychological flexibility. Cronbach’s *α* values for the Chinese versions were 0.93 and 0.95, respectively (Zhao et al., 2018). Social support was measured using the Social Support Rating Scale (SSRS), evaluating objective support, perceived social support, and utilisation of social support. Higher scores indicate greater social support. Cronbach’s α ranged from 0.89 to 0.94 (Xiao, 1994).

### 2.7 Process evaluation

A questionnaire was administered by researcher B after the intervention to assess participants’ satisfaction with the content, format, and facilitator of the intervention, as well as self-participating ratings, preferences for content and format, and willingness to continue using the strategies learned.

### 2.8 Data analysis

Data analysis followed the intention-to-treat principle. Data were double-entered by two independent researchers and analysed using IBM SPSS Version 23.0. All statistical tests were two-sided with a significance level of 0.05. Normality was assessed using the Shapiro-Wilk test and Q-Q plots. Between-group baseline differences were examined using independent samples *t*-tests, *χ^2^* tests, Fisher’s exact tests, and McNemar tests as appropriate. Non-parametric tests were used for variables that were not normally distributed. A Generalised Estimating Equation (GEE) model was used to analyse longitudinal changes, accounting for missing data and group-by-time interactions. Bonferroni correction was applied for multiple comparisons.

A subgroup analysis was conducted among participants with baseline scores of the Edinburgh Postnatal Depression Scale >9, hypothesising greater intervention benefits for individuals at higher risk of depression (Kersting and Wagner, 2012). This subgroup analysis was conducted in accordance with the registered trial protocol [ChiCTR2100047195, Version 1.0]. Effect sizes for between-group differences at 3-month follow-up were calculated using Cohen's *d* (null effect: *d* = 0; trivial: *d* < 0.2; small: 0.2 ≤ *d* < 0.5; medium: 0.5 ≤ *d* < 0.8; large: *d* ≥ 0.8) (Cohen, 2013).

### 2.9 Ethical considerations

This study has been approved by the Ethics Committee of Xiangya School of Nursing (Approval No.: e202152). Written informed consent was obtained from all participants prior to baseline assessment and group allocation. Participation was entirely voluntary, and participants retained the right to withdraw from the study at any time without affecting their access to clinical care or services. Privacy and confidentiality were rigorously maintained. All identifiable personal information and study-related data were securely stored in a password-protected cloud-based folder, accessible only to authorised research personnel. As WeChat was used for intervention delivery, participants were advised not to share sensitive information in group chats and were informed of potential privacy risks associated with this platform. Future use in other settings should consider local data protection regulations.

## 3 Results

**Fig. 1:**
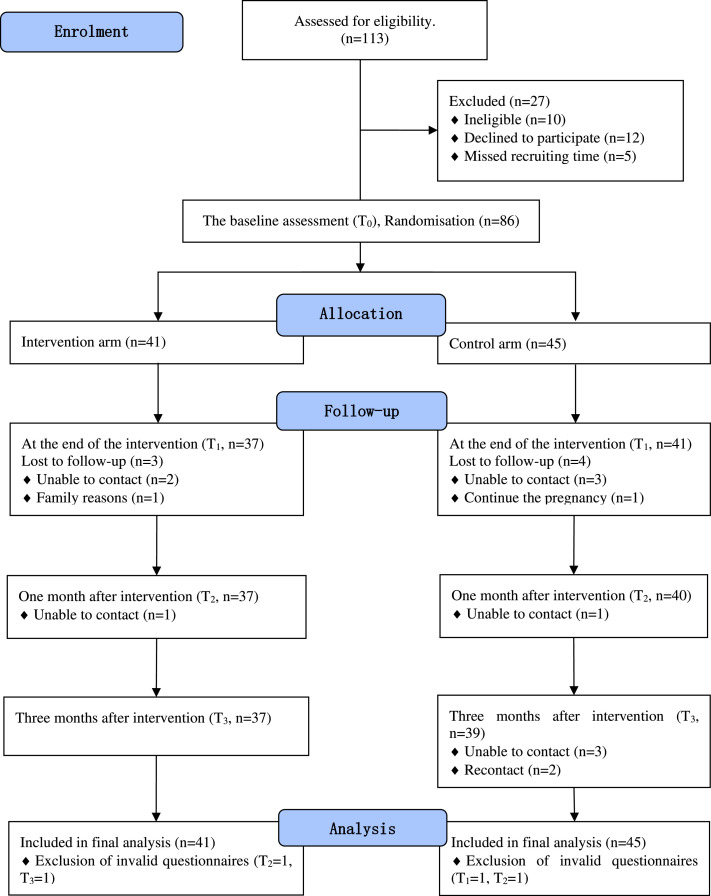
CONSORT flow diagram of recruitment.

Participant recruitment took place between August 2021 and January 2022. A total of 468 women underwent eligibility assessment, with 113 (24.15 %) women meeting our eligibility criteria. Of these, 27 (23.89 %) were excluded for the following reasons: not meeting inclusion criteria (*n* = 10), declining to participate (*n* = 12), or missing the recruitment window (*n* = 5). The remaining 86 participants (76.11 % of those eligible) completed the baseline assessment (T0) and were subsequently randomised into the intervention group (*n* = 41) or the control group (*n* = 45). The process is documented in a CONSORT flow diagram (Fig. 1).

All participants in the intervention group completed the intended intervention. Follow-up data collection was completed by April 2022. The trial concluded as originally planned without early termination or extension. Attrition rates were 8.14 % at T1, 10.47 % at T2, and 11.63 % at T3, respectively. Attrition causes in the intervention group primarily included loss of contact and familial dissent, whereas in the control group, attrition was primarily due to loss of contact. An exclusion from the control group occurred due to a participant's decision to proceed with pregnancy. No significant variance was observed in baseline characteristics between participants who completed the study and those who withdrew.

Despite these fluctuations, all 86 participants were included in the final analysis, with full data sets from 41 participants in the intervention group and 45 in the control group. One questionnaire was excluded at T1 and another at T3 in both groups due to being invalid, defined as a repetition rate of >90 % (over 90 % of items were marked with the same response option, regardless of the meaning or content of the option).

### 3.1 Demographic characteristics

Baseline demographic, clinical, and outcome-related characteristics for both the intervention (*n* = 41) and control (*n* = 45) groups are presented in Table 1. No statistically significant differences were found between the groups across all variables, indicating successful randomisation.

**Table 1:** Baseline data of demographic characteristics, clinical characteristics, and outcome variables.

Variables	Total n (%) or mean (SD)	I (*n* = 41)	C (*n* = 45)	*p-value*
Age, years; mean (SD)	29.94 (4.97)	30.27 (5.19)	29.64 (4.81)	0.564^a^
Residence, n (%)				0.384^b^
Urban	59 (68.6)	30 (73.2)	29 (64.4)	
Rural	27 (31.4)	11 (26.8)	16 (35.6)	
Educational level, n (%)				0.746^c^
Primary school and below	4 (4.7)	3 (7.3)	1 (2.2)	
Junior high school	21 (24.4)	10 (24.4)	11 (24.4)	
Senior high school	27 (31.4)	14 (34.1)	13 (28.9)	
Undergraduate	30 (34.8)	12 (29.3)	18 (40.0)	
Master and above	4 (4.7)	2 (4.9)	2 (4.4)	
Employment status, n (%)				0.253^c^
Employment	42 (48.8)	21 (51.3)	21 (46.7)	
Unemployed	11 (12.8)	6 (14.6)	5 (11.1)	
Others	33 (38.4)	14 (34.1)	19 (42.2)	
Monthly income, n (%)				0.146^c^
<3000 RMB	7 (8.1)	5 (12.2)	2 (4.4)	
3000–5000 RMB	22 (25.6)	13 (31.7)	9 (20.0)	
>5000 RMB	57 (66.3)	23 (56.1)	34 (75.6)	
Insurance, n (%)				0.578^b^
BMIUW	38 (44.2)	16 (39.0)	22 (48.9)	
NRCMI	13 (15.1)	6 (14.6)	7 (15.6)	
Self-paying	35 (40.7)	19 (46.3)	16 (35.6)	
Religion, n (%)				1.000^c^
Yes	3 (3.5)	1 (2.4)	2 (4.4)	
No	83 (96.5)	40 (97.6)	43 (95.6)	
Pregnancy times, n (%)				0.636^c^
1	30 (34.9)	15(36.6)	15 (33.3)	
2	25 (29.1)	10 (24.4)	15 (33.3)	
3 and more	31 (36.0)	16 (39.0)	15 (33.3)	
Existing children, n ( %)				0.942^c^
0	43 (50.0)	20 (48.8)	23 (51.1)	
1	38 (44.2)	19 (46.3)	19 (42.2)	
2 or more	5 (5.8)	2 (4.9)	3 (6.7)	
Gestational weeks, M (IQR)	22.28 (5.31)	23 (8)	14 (6)	0.071^d^
History of abnormal pregnancy, n ( %)				0.142^c^
Yes	20 (23.3)	9 (22.0)	11 (24.4)	
No	66 (76.7)	32 (78.0)	34 (75.6)	
Recent negative life events, n ( %)				0.347^c^
Yes	16 (18.6)	11 (26.8)	5 (11.1)	
No	70 (81.4)	30 (73.2)	40 (88.9)	
Edinburgh Postnatal Depression Scale	12.47 (5.50)	12.61 (5.25)	12.33 (5.78)	0.818
Impact of Event Scale-Revised	39.17 (15.89)	38.98(15.94)	39.36 (16.03)	0.913
Intrusive symptoms	14.90 (5.98)	14.56 (5.54)	15.20 (6.41)	0.624
Avoidance	14.90 (6.23)	14.85 (6.56)	14.96 (5.99)	0.940
Hyperarousal	9.37 (5.31)	9.56 (5.46)	9.20 (5.23)	0.755
Psychological flexibility	55.91 (18.45)	55.61 (17.39)	56.18 (19.56)	0.887
Acceptance and Action Questionnaire-II	21.95 (8.24)	22.32 (7.53)	21.62 (8.91)	0.699
Cognitive Fusion Questionnaire-Fusion	33.95 (11.36)	33.29 (10.99)	34.56 (11.78)	0.610
Social Support Rating Scale	43.55 (7.09)	43.80 (7.10)	43.47 (7.16)	0.827
Objective Support	10.60 (2.56)	2.56 (2.45)	10.82 (2.47)	0.600
Perceived Social Support	24.70 (4.52)	25.02 (4.50)	24.40 (4.57)	0.525
Utilisation of Social Support	8.24 (1.69)	8.24 (1.68)	8.24 (1.71)	0.999

Notes: I: Intervention group; C: Control group.

a *t*-test.

b Pearson chi-square test.

c Fisher exact test; RMB: Chinese dollar; BMIUW: Basic Medical Insurance for Urban Workers is a social insurance system to compensate workers for economic losses due to disease risks; NRCMI: New Rural Cooperative Medical Insurance refers to the peasant medical mutual assistant system that is guided and supported by the government and voluntarily participated by peasant.

d Mann-Whitney U test.

The mean age of the participants was 29.94 years (standard deviation, SD = 4.97), with a range of 19 to 44 years. Most participants resided in urban areas (68.6 %) and had received senior high school education (12 years of formal education) or above (70.9 %). Employment rates were 87.2 %, as were monthly incomes, with the majority earning >5000 RMB (66.3 %). More than half of the participants (59.3 %) had basic medical insurance. The most majority participants had no religion (96.5 %). Regarding clinical characteristics, both groups were similar in pregnancy history, gestational age at intervention, and the presence of existing children. The mean gestational weeks was 22.28 (SD = 5.31), ranging from 12 to 38. As gestational weeks were not normally distributed, they are reported as median and interquartile range (IQR): 23 weeks (IQR = 8) in the intervention group and 14 weeks (IQR = 6) in the control group. No significant difference was observed (*p* = 0.071, Mann–Whitney U test). A minority of participants had a history of abnormal pregnancies (23.3 %) or reported experiencing recent negative life events (18.6 %).

Baseline psychological distress levels were comparable. Mean scores of the Edinburgh Postnatal Depression Scale were 12.61 (SD = 5.25) in the intervention group and 12.33 (SD = 5.78) in the control group (*p* = 0.818). The scores of the Impact of Event Scale-Revised and its subdomains—intrusion, avoidance, and hyperarousal—also showed no significant differences. Measures of psychological flexibility (the Acceptance and Action Questionnaire-II and the Cognitive Fusion Questionnaire-Fusion), and social support (the Social Support Rating Scale and its subdomains) were balanced between the two groups (all *p* > 0.05).

These findings confirm that the two groups were well matched at baseline in terms of sociodemographic, obstetric, and psychological characteristics.

### 3.2 Outcomes and estimation

The results of the Generalised Estimating Equation models assessing changes in primary and secondary outcomes over time are summarised in Table 2.

**Table 2:** Generalised estimating equation model for the comparison of outcome variables between groups across the study period.

	I	C	Time effect		Group effect		Group*Time effect		Cohen's *d*
	Mean (SD)	Mean (SD)	*β* (95 %CI)	*p-value*	*β* (95 %CI)	*p-value*	*β* (95 %CI)	*p-value*	
**Edinburgh Postnatal Depression Scale**	.91 (−0.66, 2.48)	.254		.887					
T_0_	12.61 (5.25)	12.33 (5.78)					.28 (−2.10, 2.65) *	.818*	
T_1_	9.68 (3.54) a	10.90 (5.55) a	2.19 (−0.65, 5.03)	.654			1.10 (−0.21, 2.40) #	.099#	
T_2_	9.08 (4.34) a	10.10 (6.78) a	1.45 (−1.39, 4.28)	.318			.72 (−1.10, 2.54) #	.438#	
T_3_	8.25 (5.06) a	9.62 (6.79) a	.71 (−2.41, 3.84)	.130			.92 (−1.38, 3.21) #	.435#	0.30
**Impact of Event Scale-Revised**	4.33 (−0.76, 9.41)	.095		.424					
T_0_	38.98 (15.94)	39.36 (16.03)					−0.38 (−7.24, 6.48) *	.913*	
T_1_	36.68 (12.61)	38.67 (15.46)	15.73 (5.72, 25.74)	.002			2.34 (−2.29, 6.97) #	.322#	
T_2_	29.22 (14.46) a	34.44 (17.48)	9.75 (−0.26, 19.76)	.056			5.43 (−0.52, 11.38) #	.073#	
T_3_	25.03 (13.72) a	31.62 (19.22) a	5.47 (−4.36, 15.31)	.276			5.31 (−1.25, 11.86) #	.113#	0.39
Intrusion			1.09 (−0.82, 3.01)	.263		.783			
T_0_	14.56 (5.54)	15.20 (6.41)					−0.64 (−3.22, 1.94) *	.624*	
T_1_	13.78 (4.80)	14.73 (6.06)	6.56 (2.73, 10.39)	.001			.75 (−1.07, 2.57) #	.418#	
T_2_	10.75 (5.01) a	12.15 (6.90) a	3.72 (−0.08, 7.51)	.055			1.12 (−1.20, 3.43) #	.344#	
T_3_	9.08 (4.74) a	11.36 (7.31) a	2.20 (−1.52, 5.93)	.246			1.43 (−1.00, 3.86) #	.250#	0.27
Avoidance			2.27 (0.26, 4.27)	.027		.022			
T_0_	14.85 (6.56)	14.96 (5.99)					−0.10 (−2.79, 2.59) *	.940*	
T_1_	14.68 (5.09)	15.03 (5.68)	7.67 (3.88, 11.47)	.000			.66 (−1.36, 2.68) #	.521#	
T_2_	10.83 (5.59) a	13.69 (6.07)	5.06 (1.44, 8.69)	.006			3.24 (1.00, 5.48) #	.005#	
T_3_	9.61 (5.57) a	12.82 (7.23) a	3.70 (0.04, 7.36)	.048			2.98 (0.27, 5.70) #	.031#	0.49
Hyperarousal				.97 (−0.53, 2.47)	.205		.980		
T_0_	9.56 (5.46)	9.20 (5.23)					.36 (−1.93, 2.65) *	.755*	
T_1_	8.22 (4.37)	8.93 (4.94)	2.81 (0.82, 4.79)	.006			.91 (−0.56, 2.38) #	.361#	
T_2_	7.64 (4.92) a	8.59 (5.70)	2.29 (0.19, 4.39)	.032			1.07 (−0.80, 2.93) #	.262#	
T_3_	6.33 (4.52) a	7.44 (6.06) a	.92 (−1.16, 3.00)	.385			.94 (−1.07, 2.95) #	.225#	0.25
**Psychological Flexibility**	.30 (−5.48, 6.08)	.920		.922					
T_0_	55.61 (17.39)	56.18 (19.56)				−0.57 (−8.54, 7.40) *	.888*		
T_1_	56.30 (14.46)	57.43 (20.61)	22.21 (8.14, 36.29)	.002		.37 (−4.81, 5.55) #	.889#		
T_2_	53.22 (15.63)	55.49 (21.93)	19.77 (6.14, 33.40)	.004		1.13 (−5.65, 7.90) #	.745#		
T_3_	48.61 (20.43) a	49.97 (26.46)	14.28 (−0.60, 29.17)	.060		−0.59 (−9.79, 8.62) #	.901#	0.04	
Acceptance and Action Questionnaire-II	.42 (−2.22, 3.06)	.757		.926					
T_0_	22.32 (7.53)	21.62 (8.91)				.69 (−2.86, 4.25) *	.699*		
T_1_	22.59 (6.28)	23.05 (9.10)	9.34 (3.83, 14.85)	.001		.67 (−1.59, 2.92) #	.562#		
T_2_	21.89 (7.16)	22.36 (9.29)	8.67 (3.14, 14.19)	.002		.59 (−2.48, 3.66) #	.705#		
T_3_	19.39 (8.89) a	19.59 (11.99)	5.86 (−0.14, 11.85)	.055		−0.01 (−4.26, 4.24) #	.996#	0.11	
Cognitive Fusion Questionnaire-Fusion	−0.02 (−3.49, 3.44)	.989		.904					
T_0_	33.29 (10.99)	34.56 (11.78)				−1.26 (−6.16, 3.64) *	.610*		
T_1_	33.70 (9.26)	34.38 (12.46)	14.80 (6.96, 22.65)	.000		−0.22 (−3.77, 3.33) #	.904#		
T_2_	31.33 (9.20)	33.13 (13.58)	13.03 (5.66, 20.41)	.001		.63 (−3.59, 4.84) #	.770#		
T_3_	29.22 (12.31) a	30.38 (15.20)	10.36 (2.27, 18.46)	.012		−0.46 (−5.81, 4.88) #	.865#	0.01	
**Social Support Rating Scale**	−1.79 (−3.84, 0.25)	.086		.163					
T_0_	43.68 (7.10)	43.42 (7.15)				.26 (−2.80, 3.32) *	.866*		
T_1_	43.95 (6.47)	42.20 (8.06) a	6.41 (−2.02, 14.84)	.136		−0.79 (−2.64, 1.06) #	.401#		
T_2_	44.56 (6.83)	41.26 (9.40) a	6.18 (−2.19, 14.55)	.148		−2.12 (−4.59, 0.36) #	.094#		
T_3_	44.47 (7.30)	41.00 (8.44) a	5.91 (−2.58, 14.40)	.173		−2.51 (−5.36, 0.34) #	.085#	0.45	
Objective Support		.04 (−0.91, 0.98)	.940		.726				
T_0_	10.41 (2.68)	10.78 (2.47)				−0.36 (−1.47, 0.74) *	.515*		
T_1_	10.78 (2.58)	11.05 (2.90)	3.61 (0.83, 6.40)	.011		.27 (−0.63, 1.18) #	.556#		
T_2_	10.89 (3.00)	10.92 (3.42)	3.56 (0.82, 6.30)	.011		.06 (−1.05, 1.16) #	.920#		
T_3_	10.86 (3.21)	10.69 (3.61)	3.39 (0.59, 6.20)	.018		−0.22 (−1.68, 1.23) #	.763#	0.21	
Perceived Social Support		−1.30 (−2.44, −0.17)	.025		.408				
T_0_	25.02 (4.50)	24.40 (4.57)				.62 (−1.32 (2.57) *	.525*		
T_1_	25.03 (3.91)	23.48 (4.97) a	5.91 (1.87, 9.95)	.004		−0.77 (−1.84, 0.30) #	.159#		
T_2_	25.39 (3.80)	22.87 (5.45) a	5.78 (1.90, 9.67)	.004		−1.60 (−3.23, 0.03) #	.054#		
T_3_	25.44 (4.02)	23.08 (4.71) a	5.86 (1.88, 9.85)	.004		−1.56 (−3.10, −0.02) #	.047#	0.38	
Utilisation of Social Support	−0.62 (−1.12, −0.13)	.013		.280					
T_0_	8.24 (1.68)	8.24 (1.71)				.00 (−0.73, 0.73) *	.999*		
T_1_	8.14 (1.93)	7.68 (1.64) a	2.19 (0.91, 3.46)	.003		−0.37 (−0.90, 0.15) #	.161#		
T_2_	8.28 (1.85)	7.46 (2.10) a	2.14 (0.87, 3.41)	.001		−0.68 (−1.34, −0.02) #	.044#		
T_3_	8.17 (1.86)	7.23 (1.80) a	1.95 (0.68, 3.23)	.001		−0.83 (−1.48, −0.18) #	.013#	0.55	

Note: I: Intervention group; C: Control group. T_0_: Baseline (*n* = 41/45), T_1_: 1-week (*n* = 37/40), T_2_: 1-Month (*n* = 36/39), T_3_: 3-Months (*n* = 36/39). *β*: regression coefficient.

a *p* < 0.05 compared to T_0_.

⁎ Based on the *t*-test.

# Independent effects of interventions based on Generalised Estimating Equation obtained after correcting for baseline.

#### 3.2.1 Primary outcomes

Depressive symptoms, as measured by the Edinburgh Postnatal Depression Scale, decreased over time in both groups. Although no statistically significant group-by-time interaction was observed (*β* = 0.92, 95 % CI: −1.38 to 3.21, *p* = 0.435), the intervention group consistently showed lower mean scores of the Edinburgh Postnatal Depression Scale than the control group at all follow-up points. The between-group effect size at three months post-intervention was small (*d* = 0.30).

Posttraumatic stress symptoms measured by the Impact of Event Scale-Revised also declined across the study period. While the overall group-by-time interaction was not statistically significant (*β* = 5.31, 95 % CI: −1.25 to 11.86, *p* = 0.113), the intervention group showed a greater reduction in scores compared to the control group, with a small effect size at three months (*d* = 0.39). Among the subscales of the Impact of Event Scale-Revised, statistically significant group-by-time interaction effects were observed for avoidance (*β* = 2.98, 95 % CI: 0.27 to 5.70, *p* = 0.031; *d* = 0.49), whereas intrusion and hyperarousal symptoms showed no statistically significant differences.

#### 3.2.2 Secondary outcomes

Psychological flexibility increased over time in both groups. No statistically significant group-by-time interaction was found for total scores (*β* = −0.59, 95 % CI: −9.79 to 8.62, *p* = 0.901; *d* = 0.04) or acceptance (*d* = 0.11) and cognitive fusion subscales (*d* = 0.01).

In contrast, improvements were more pronounced in social support domains. Statistically significant group-by-time interactions were observed for utilisation of social support (*β* = −0.83, 95 % CI: −1.48 to −0.18, *p* = 0.013), with a medium effect size (*d* = 0.55). Additionally, statistically significant improvements in perceived social support were observed in the intervention group over time (*β* = −1.56, 95 % CI: −3.10 to −0.02, *p* = 0.047; *d* = 0.38), although differences in objective social support did not reach statistical significance (*p* = 0.763, *d* = 0.21).

### 3.3 Sub-group analysis

A predefined subgroup analysis was conducted among participants who had elevated depressive symptoms at baseline (the score of the Edinburgh Postnatal Depression Scale > 9), to examine whether the intervention had a more pronounced effect in this high-risk population. A total of 54 participants (intervention group: *n* = 31; control group: *n* = 23) met the inclusion criteria for this analysis. The results of subgroup analysis are summarised in Table 3.

**Table 3:** Generalised estimating equation model for sub-analysis.

	I	C	Time effect		Group effect		Group*Time effect		Cohen’s *d*
	Mean (SD)	Mean (SD)	*β* (95 %CI)	*p* value	*β* (95 %CI)	*p* value	*β* (95 %CI)	*p* value	
**Edinburgh Postnatal Depression Scale**	1.83 (−0.19, 3.85)	.076		.757					
T_0_	14.77 (3.91)	17.04 (4.11)					−2.27 (−4.48, −0.06) *	.044*	
T_1_	10.59 (3.43) a	14.43 (4.96) a	.22 (−3.74, 4.18)	.913			2.02 (0.38, 3.66) #	.016#	
T_2_	10.31 (4.00) a	13.57 (6.86) a	−0.31 (−4.29, 3.67)	.878			1.43 (−1.04, 3.90) #	.258#	
T_3_	8.58 (3.85) a	12.71 (7.11) a	−1.78 (−5.77, 2.22)	.383			2.03 (−0.68, 4.74) #	.142#	0.46
**Impact of Event Scale-Revised**		9.51 (2.40, 16.62)	.009		.406				
T_0_	44.45 (13.71)	49.09 (15.39)					−4.64 (−12.61, 3.34) *	.249*	
T_1_	39.33 (10.60) a	48.00 (13.94)	19.08 (3.63, 34.53)	.016			7.28 (1.41, 13.15) #	.015#	
T_2_	31.77 (14.17) a	42.43 (18.09)	12.25 (−3.50, 27.99)	.127			9.55 (1.41, 17.69) #	.022#	
T_3_	26.27 (13.83) a	39.90 (21.27) a	7.70 (−7.78, 23.19)	.330			11.75 (2.39, 21.12) #	.014#	0.61
Intrusion		3.37 (0.48, 6.26)	.022		.376				
T_0_	16.16 (5.08)	19.09 (6.16)					−2.93 (−6.00, 0.15) *	.062*	
T_1_	14.78 (4.19)	18.33 (5.49)	9.09 (3.12, 15.06)	.003			2.77 (0.28, 5.26) #	.029#	
T_2_	11.31 (5.24) a	15.19 (7.37) a	5.75 (−0.27, 11.77)	.061			3.13 (−0.25, 6.51) #	.070#	
T_3_	9.38 (4.99) a	14.62 (8.15) a	4.30 (−1.61, 10.22)	.154			4.22 (0.56, 7.89) #	.024#	0.41
Avoidance		3.94 (1.38, 6.51)	.003		.295				
T_0_	16.61 (6.09)	17.57 (6.53)					−0.95 (−4.47, 2.56) *	.588*	
T_1_	15.22 (3.98)	18.19 (5.33)	8.07 (3.61, 12.52)	.000			2.81 (0.58, 5.04) #	.013#	
T_2_	11.69 (5.47) a	15.95 (6.34)	5.02 (0.63, 9.41)	.025			4.27 (1.41, 7.14) #	.003#	
T_3_	10.19 (5.67) a	15.14 (7.85)	3.73 (−0.86, 8.32)	.111			4.77 (1.05, 8.50) #	.012#	0.63
Hyperarousal			2.33 (0.25, 4.41)	.028		.607			
T_0_	11.68 (4.34)	12.43 (4.87)					−0.76 (−3.28, 1.77) *	.550*	
T_1_	9.33 (4.19) a	11.48 (4.85)	2.36 (−0.70, 5.42)	.131			1.83 (−0.20, 3.86) #	.078#	
T_2_	8.77 (4.71) a	11.29 (5.75)	1.92 (−1.28, 5.12)	.239			2.27 (−0.19, 4.73) #	.070#	
T_3_	6.69 (4.52) a	10.14 (6.70) a	.12 (−2.98, 3.21)	.941			2.90 (0.18, 5.61) #	.037#	0.58
**Psychological Flexibility**	5.75 (−1.17, 12.67)	.103		.864					
T_0_	61.48 (14.42)	67.00 (17.92)				−5.52 (−14.35, 3.32) *	.216*		
T_1_	60.74 (11.33)	68.38 (19.66)	21.52 (5.48, 37.56)	.009		4.53 (−0.98, 10.05) #	.107#		
T_2_	56.81 (15.00)	65.71 (21.16)	18.24 (1.62, 34.86)	.031		5.63 (−2.87, 14.13) #	.194#		
T_3_	49.88 (17.21) a	61.90 (27.56)	11.97 (−4.25, 28.20)	.148		7.13 (−3.81, 18.07) #	.201#	0.40	
Acceptance and Action Questionnaire-II	2.49 (−0.64, 5.62)	.118		.913					
T_0_	24.74 (6.57)	26.78 (8.25)				−2.04 (−6.09, 2.00) *	.316*		
T_1_	24.89 (4.57)	28.48 (7.93)	10.84 (3.88, 17.80)	.002		2.54 (0.30, 4.78) #	.026#		
T_2_	23.54 (6.72)	26.67 (9.19)	9.29 (2.19, 16.40)	.010		2.06 (−1.67, 5.80) #	.279#		
T_3_	20.04 (7.11) a	24.57 (12.85)	6.15 (−0.94, 13.25)	.089		2.87 (−2.25, 8.00) #	.272#	0.34	
Cognitive Fusion Questionnaire-Fusion	3.56 (−0.89, 8.00)	.117		.715					
T_0_	36.74 (9.48)	40.22 (10.60)				−3.48 (−8.98, 2.03) *	.211*		
T_1_	35.85 (7.81)	39.90 (12.81)	13.74 (5.25, 22.22)	.002		2.27 (−2.00, 6.54) #	.298#		
T_2_	33.27 (8.77)	39.05 (12.92)	12.00 (3.32, 20.67)	.007		3.85 (−1.57, 9.28) #	.164#		
T_3_	29.85 (11.11) a	37.33 (15.69)	8.89 (0.54, 17.24)	.037		4.59 (−2.00, 11.18) #	.172#	0.40	
**Social Support Rating Scale**	−2.23 (−4.95, 0.48)	.107		.416					
T_0_	42.58 (7.15)	40.91 (6.42)				1.67 (−2.12, 5.45) *	.381*		
T_1_	43.04 (6.67)	40.14 (6.92)	10.23 (−1.47, 21.93)	.086		−1.27 (−3.80, 1.27) #	.327#		
T_2_	43.62 (7.01)	39.48 (8.52)	10.17 (−1.34, 21.67)	.083		−2.35 (−5.66, 0.96) #	.165#		
T_3_	43.88 (7.33)	38.43 (8.00)	10.10 (−1.73, 21.93)	.094		−3.12 (−6.91, 0.68) #	.107#	0.54	
Objective Support		−0.27 (−1.51, 0.98)	.674		.501				
T_0_	9.84 (2.60)	10.13 (2.20)				−0.29 (−1.64, 1.05) *	.665*		
T_1_	10.33 (2.51)	10.10 (2.32)	5.56 (2.09, 9.03)	.002		−0.18 (−1.34, 0.98) #	.766#		
T_2_	10.42 (3.04)	10.57 (3.31)	5.80 (2.44, 9.17)	.001		−0.18 (−1.34, 0.98) #	.740#		
T_3_	10.46 (3.09)	9.48 (3.68)	5.35 (1.72, 8.97)	.004		−0.87 (−2.81, 1.07) #	.380#	0.51	
Perceived Social Support		−1.33 (−3.01, 0.35)	.121		.681				
T_0_	24.68 (4.69)	22.83 (4.01)				1.85 (−0.59, 4.29) *	.134*		
T_1_	24.78 (4.07)	22.57 (4.44)	6.64 (0.58, 12.70)	.032		−0.84 (−2.46, 0.77) #	.306#		
T_2_	24.96 (4.11)	21.81 (5.05)	6.37 (0.75, 11.98)	.026		−1.73 (−4.27, 0.82) #	.183#		
T_3_	25.38 (4.29)	22.14 (4.48)	6.92 (0.90, 12.94)	.024		−1.43 (−3.39, 0.53) #	.151#	0.31	
Utilisation of Social Support		−0.74 (−1.40, −0.07)	.029		.024				
T_0_	8.06 (1.63)	7.96 (1.82)				.11 (−0.84, 1.05) *	.820*		
T_1_	7.93 (2.06)	7.48 (1.54)	2.23 (0.65, 3.80)	.006		−0.31 (−1.03, 0.40) #	.392#		
T_2_	8.23 (1.99)	7.10 (2.00) a	2.21 (0.59, 3.82)	.007		−0.96 (−1.72, −0.20) #	.013#		
T_3_	8.04 (1.95)	6.81 (1.69) a	2.02 (0.45, 3.60)	.012		−0.95 (−1.85, −0.04) #	.040#	0.65	

Note: I: Intervention group; C: Control group. T_0_: Baseline (*n* = 31/23), T_1_: 1-week (*n* = 27/21), T_2_: 1-Month (*n* = 26/21), T_3_: 3-Months (*n* = 26/21). *β*: regression coefficient;.

a *p*<0.05 compared to T_0_.

⁎ Based on the *t*-test.

# Independent effects of interventions based on Generalised Estimating Equation obtained after correcting for baseline.

Among these participants, the intervention group demonstrated a statistically significant reduction in depressive symptoms compared to the control group at T1 (*β* = 2.02, 95 % CI: 0.38 to 3.66, *p* = 0.016). The group-by-time interaction effect for the Edinburgh Postnatal Depression Scale was marginally significant (*β* = 2.03, 95 % CI: −0.68 to 4.74, *p* = 0.142), with a small effect size at three months (*d* = 0.46).

More notably, the intervention group showed statistically significant improvements in post-traumatic stress symptoms, including intrusion, avoidance and hyperarousal symptoms. The group-by-time interaction for the total score of the Impact of Event Scale-Revised was statistically significant (*β* = 11.75, 95 % CI: 2.39 to 21.12, *p* = 0.014), with a medium effect size (*d* = 0.61). Intrusive symptoms (*β* = 4.22, *p* = 0.024; *d* = 0.41), avoidance symptoms (*β* = 4.77, *p* = 0.012; *d* = 0.63), and hyperarousal symptoms (*β* = 2.90, *p* = 0.037; *d* = 0.58) all showed statistically significant improvements in the intervention group.

Secondary outcomes also favoured the intervention group. Experiential avoidance significantly improved at T1 (*β* = 2.54, 95 % CI: 0.30 to 4.78; *p* = 0.026). Utilisation of social support showed statistically significant improvements over time in the intervention group compared to the control group (*β* = −0.95, 95 % CI: −1.85 to −0.04; *p* = 0.040), with a medium effect size (*d* = 0.65). Improvements in psychological flexibility, cognitive fusion, and perceived social support were observed but did not reach statistical significance.

### 3.4 Process evaluation

Twenty-nine participants in the intervention group completed a satisfaction survey, and the results indicate high levels of satisfaction with the intervention's content, format, and facilitators (Table 4). Nearly half (48.28 %) found the unit 2 *‘Creative Hopelessness, Defusion and Acceptance’* most useful, followed by unit 3 *‘Living in the Present’* and unit 4 *‘Clarifying Values and Committing to Action’*, each favoured by 20.69 %. The vast majority (96.55 %) expressed a willingness to continue using the strategies they had learned. While 75.86 % preferred in-person intervention, others favoured online videos due to privacy concerns. The mean acceptability score for the online intervention was 7.72 out of 10 (SD = 1.58), and the mean self-rated engagement score was 8.29 out of 10 (SD = 1.03).

**Table 4:** Results of the satisfaction questionnaire.

Items	*n* = 29	%
Satisfaction with the content of the intervention		
Strongly Satisfy	19	65.52
Satisfy	10	34.48
Neither satisfied nor dissatisfied	0	0
Dissatisfy	0	0
Strongly dissatisfy	0	0
Satisfaction with the format of the intervention		
Strongly Satisfy	20	68.97
Satisfy	8	27.59
Neither satisfied nor dissatisfied	1	3.45
Dissatisfy	0	0
Strongly dissatisfy	0	0
Satisfaction with interventionists		
Strongly Satisfy	28	96.6
Satisfy	1	3.4
Neither satisfied nor dissatisfied	0	0
Dissatisfy	0	0
Strongly dissatisfy	0	0
The most helpful units of Acceptance and Commitment Therapy		
Unit 1: A Brief Introduction to Acceptance and Commitment Therapy and Mindfulness Breathing	3	10.34
Unit 2: Creative Hopelessness, Defusion and Acceptance	14	48.28
Unit 3: Living in the Present - Mindfulness Practice	6	20.69
Unit 4: Clarifying Values and Committing to Action	6	20.69
Intention to continue using the strategies		
Yes	28	96.55
No	1	3.45
Preference for intervention format		
In-person	22	75.86
Online	7	24.14
Self-raking involvement, Mean (SD), Range	8.29 (1.03)	6 - 10
Acceptability for online intervention, Mean (SD), Range	7.72 (1.58)	3 - 10

## 4 Discussion

This study evaluated the effectiveness of the multi-component psychosocial intervention programme in improving the psychological well-being of women undergoing termination of pregnancy for foetal anomaly. Overall, this programme demonstrated positive effects on reducing psychological distress and enhancing social support among women undergoing termination of pregnancy for foetal anomaly, particularly those at higher risk for depressive symptoms. While not all group-by-time interactions reached statistical significance in the full sample, the intervention group consistently showed favourable trends in reduced depressive and post-traumatic stress symptoms, as well as improved utilisation and perception of social support. However, these differences should be interpreted with caution due to the lack of statistical significance. Subgroup analyses further revealed more substantial effects among participants with elevated baseline depressive symptoms, supporting that the intervention may be particularly beneficial for women at high risk of psychological distress following termination of pregnancy for foetal anomaly. No adverse events were reported throughout the study period, indicating the safety and acceptability of the intervention.

A multi-component intervention approach has been suggested that can more effectively address the complex needs of women undergoing termination of pregnancy for foetal anomaly (Zhou et al., 2023). Our results showed that participants in the intervention group experienced a greater reduction in post-traumatic stress disorder-related avoidance symptoms compared to controls. The subgroup analysis further revealed that small-to-moderate effect sizes in alleviating depressive symptoms and post-traumatic stress symptoms among women with baseline scores of the Edinburgh Postnatal Depression Scale above 9, supporting the effectiveness of the multi-component psychosocial intervention programme in mitigating psychological distress for high-risk populations. These findings align with the findings of Pedraza et al. (2024), which underscore the importance of comprehensive psychosocial interventions for bereaved parents. The multi-component psychosocial intervention programme, which integrates informational support, Acceptance and Commitment Therapy, online peer support groups and family support, may have fostered a sense of support and facilitated adaptive coping strategies, contributing to the observed psychological benefits.

However, professional psychological support may not universally be required by women undergoing termination of pregnancy for foetal anomaly. No statistically significant differences were observed in improving depression and post-traumatic stress symptoms amongst the total sample. We assume that this may be because the multi-component psychosocial intervention programme was developed based on the needs of most women undergoing termination of pregnancy for foetal anomaly (Wang et al., 2024), while for some women without psychological distress, the multiple components may cause a burden. However, the participants with depressive symptoms in the intervention group showed a significant reduction in both depression and post-traumatic stress symptoms, which suggested that women undergoing termination of pregnancy for foetal anomaly with depressive symptoms are more likely to benefit from the structured multi-component psychosocial intervention programme. These results are consistent with prior studies, which demonstrated that the participants who are more aware of and suffer more from negative feelings are more likely to benefit from professional mental health support (Qian et al., 2021). Similar results were also reported by Bittner et al. (2014), who found that women with elevated depressive symptoms experienced greater improvement from professional psychological intervention compared to those with lower symptoms. This may also indicate that not every woman undergoing termination of pregnancy for foetal anomaly needs all components in this programme, especially those women without depressive symptoms. Therefore, we recommend that clinical practitioners should pay attention to identifying women undergoing termination of pregnancy for foetal anomaly with psychological distress and provide the intervention components according to the individual’s needs and mental health conditions. Meanwhile, a flexible approach to implement the intervention components should be explored in future research to ensure the adaptability and effectiveness of the multi-component psychosocial intervention programme.

Improvements in perceived social support and utilisation of social support further suggest that the blended model of face-to-face and digital support can enhance women's coping resources. Our findings indicate that women undergoing termination of pregnancy for foetal anomaly in the control group experienced a significant decline in social support, likely due to the absence of structured support mechanisms. In contrast, participants in the intervention group reported significantly higher levels of perceived social support and greater utilisation of social support compared to those in the control group. These results align with previous research showing that support from medical staff and family members plays a crucial role in enhancing social support for women undergoing termination of pregnancy for foetal anomaly (Sun et al., 2018). This effect in our study may be partially attributed to the health education materials provided by the research team, which likely facilitated a better understanding of the termination of pregnancy for foetal anomaly among family members. Additionally, experiences shared in the online peer support group were associated with increased utilisation of support from medical professionals, families, and peers (Jieqiong et al., 2018). Meanwhile, the skills based on Acceptance and Commitment Therapy may help women live in the moment, leading to an increased perception of available support. The emotional support provided by intervention personnel and peers may have further contributed to a heightened sense of being supported, reinforcing the psychological benefits of the intervention.

Furthermore, our findings highlighted the effectiveness of the multi-component psychosocial intervention programme in reducing avoidance symptoms and fostering acceptance among women undergoing termination of pregnancy for foetal anomaly. The intervention group showed significant improvement in post-traumatic stress disorder-related avoidance symptoms compared to the control group at both T2 and T3, suggesting that the programme may have played a role in mitigating avoidance behaviours. Additionally, among participants with depression, a significant reduction in experiential avoidance, a core component of psychological flexibility, was observed immediately after the intervention, coinciding with a significant improvement in depressive symptoms. Previous research has identified avoidance and closed attitudes toward foetal anomaly as predominant coping strategies among women who have experienced termination of pregnancy for foetal anomaly (Qin et al., 2019). Acceptance and Commitment Therapy, a key component of the multi-component psychosocial intervention programme, has been recognised as a promising therapeutic approach for perinatal women with depression due to its emphasis on values, mindfulness, and acceptance (Bai et al., 2020; Bonacquisti et al., 2017). The observed reduction in avoidance symptoms, alongside improvements in psychological flexibility and depression, suggests that the intervention may facilitate a shift in coping strategies, from avoidance to acceptance, ultimately contributing to the alleviation of depressive symptoms and overall psychological adaptation.

The findings of the process evaluation suggest that the intervention was well-received by participants, demonstrating strong feasibility and acceptability within the clinical setting. High satisfaction with the intervention’s content, format, and facilitators indicates that the components were perceived as relevant and supportive in addressing participants' psychosocial needs. The fact that nearly half of the participants identified *‘Creative Hopelessness, Defusion, and Acceptance’* as the most useful unit underscores the importance of addressing emotional avoidance and fostering psychological flexibility in the context of perinatal bereavement. The relatively high endorsement of *‘Living in the Present’* and *‘Clarifying Values and Committing to Action’* further highlights participants' engagement with mindfulness and values-based approaches, core components of Acceptance and Commitment Therapy. The overwhelming willingness (96.55 %) to continue using the strategies learned suggests a strong potential for long-term impact on coping and adjustment following loss. Preferences for in-person versus online formats varied, highlighting the need for flexibility in delivery modes to accommodate individual preferences and address privacy concerns. The relatively high acceptability and engagement scores for the online component also indicate the promise of digital tools to supplement or extend in-person interventions, particularly in contexts with limited resources. Overall, these results provide preliminary support for the intervention's potential to enhance psychological resilience and emotional processing among women undergoing termination of pregnancy for foetal anomaly, highlighting the need for flexible delivery formats and further evaluation in larger samples with extended follow-up periods.

The specific cultural and health system context of this study may influence the generalizability of our findings. The research was conducted in two large maternity hospitals in Hunan Province, China, where hospital-based care is the primary mode of service delivery and psychosocial intervention is not routinely integrated into standard maternity care. In this setting, cultural attitudes toward perinatal loss are often shaped by stigma and limited openness about mental health (Li et al., 2023), which may affect both the willingness to seek support and the acceptability of psychosocial interventions. Additionally, resources for bereavement care and mental health support are often constrained compared to some Western health systems (Pedraza et al., 2024). As a result, the intervention’s effects and implementation may differ in contexts with alternative models of care, different cultural norms, or more robust psychosocial intervention infrastructures. Future studies should assess the applicability of these findings in other regions and health system settings.

### 4.1 Strengths and limitations

This study has several notable strengths. It is among the first randomised controlled trials to evaluate a structured, multi-component psychosocial intervention programme specifically designed for women undergoing termination of pregnancy for foetal anomaly (Wang et al., 2024). The intervention was theoretically grounded in the cognitive-emotional-behavioural framework of women undergoing termination of pregnancy for foetal anomaly (Qin et al., 2019) and developed based on a comprehensive understanding of the psychological and social needs of the target population (Zhou et al., 2023). The integration of both face-to-face and digital delivery formats enhanced feasibility and accessibility, especially in resource-constrained settings. Furthermore, the inclusion of a predefined subgroup analysis among women with elevated depressive symptoms allowed for deeper insights into the differential impact of the intervention.

However, some limitations must be acknowledged. First, the trial was conducted in two hospitals within a single province in China, which may limit the external validity of the findings. Second, the three-month follow-up duration may not adequately reflect the long-term effects of the programme on depression and psychological flexibility, underscoring the need for extended follow-up periods to ascertain the intervention's long-term effect. Third, the brief hospital stay limits the learning process of Acceptance and Commitment Therapy sessions, potentially reducing the measurable impact on psychological flexibility outcomes. Future studies should explore alternative delivery models, such as extending the therapy sessions over a longer period or incorporating virtual follow-up sessions to reinforce learning and practice. Finally, although subgroup analysis provided valuable insights, it may be subject to reduced statistical power and potential type I errors due to multiple comparisons.

Despite these limitations, the findings provide a valuable foundation for future research and contribute to the growing evidence base supporting tailored psychosocial care for women following termination of pregnancy for foetal anomaly.

## 5 Conclusions

This study demonstrated the effectiveness of a multi-component psychosocial intervention programme in alleviating avoidance symptoms and strengthening perceived social support and utilisation of social support. Notably, participants with depressive symptoms experienced additional benefits, with significant improvements in depression, post-traumatic stress symptoms, and utilisation of social support. Thus, we suggest healthcare providers tailor this intervention programme for women undergoing termination of pregnancy for foetal anomaly according to their individual needs and psychological conditions. Further large-scale studies are needed to confirm these results and to assess the long-term outcomes of the intervention, which offers a promising and feasible approach to enhancing bereavement care in maternity services. In addition, future research should recruit statistically powered subgroups of women with and without baseline psychological distress to enable more precise assessment of the intervention’s effects in these distinct populations.

## Funding

This study was supported by the 10.13039/501100001809National Natural Science Foundation of China (72074225); the Hunan Provincial Philosophy and Social Science Foundation (22YBA004); the Hunan Provincial Social Science Foundation23YBA011).

## CRediT authorship contribution statement

**Chunxiang Qin:** Writing – review & editing, Supervision, Methodology, Funding acquisition, Conceptualization. **Ying Li:** Writing – review & editing, Supervision, Methodology, Funding acquisition, Conceptualization. **Ying Wang:** Methodology, Investigation, Formal analysis. **Chi Huang:** Funding acquisition, Conceptualization. **Gui Xiao:** Validation, Methodology. **Lihong Zeng:** Methodology, Formal analysis. **Yanjuan He:** Investigation. **Wei Jiang:** Investigation. **Jiaying Xie:** Writing – original draft, Project administration, Methodology, Funding acquisition, Formal analysis, Data curation, Conceptualization.

## Declaration of competing interest

The authors declare that they have no known competing financial interests or personal relationships that could have appeared to influence the work reported in this paper.

## Appendix Supplementary materials

Image, application 1
