# Supplementary material for: A multi-component psychosocial intervention programme to reduce psychological distress and enhance social support for women undergoing termination of pregnancy for foetal anomaly in China: A randomised controlled trial
Source: Int J Nurs Stud Adv. 2025 Jul 29;9:100389. doi: 10.1016/j.ijnsa.2025.100389 (PMC12357282; doi:10.1016/j.ijnsa.2025.100389)
Supplement: Supplementary file 1 [file mmc1.docx]

**Supplementary material 1: Mixed Randomisation Scheme**

This study employed a mixed randomisation approach, combining simple randomisation with permuted block randomisation. Simple randomisation adds unpredictability, while permuted block randomisation ensures balance in sample sizes between groups. The mixed strategy in this study begins with the creation of an unequal block using a replacement randomisation sequence, followed by permuted blocks of variable sizes. The initial unequal block introduces sample imbalance, while the remainder of the allocation process remains unpredictable and secure. The steps of mixed randomisation^[[1]](#footnote-2)^ used in this study are as follows:

**Step 1:** The researchers determined that the first unequal block would have a block size of 8, with a predetermined allocation imbalance in which the sample sizes of Group A and Group B differ by 2.

**Step 2:** A simple random sequence of size 8 was generated using a online random number generator (<https://www.random.org/>) to implement replacement randomisation. The process was repeated until a sequence was obtained in which the difference between the number of participants in Group A and Group B was at least 2. As shown in Table 1.1, even numbers were assigned to Group A and odd numbers to Group B. The resulting allocation sequence was B, A, B, A, A, B, B, A—leading to 5 participants in Group A and 3 in Group B.

Table 1.1 Example of replacement randomisation sequence for the first unequal block (Bolock size = 8, preset group size difference = 2)

| **Trial** | **Sequence Type** | **Subject No.** | | | | | | | | **Group Size Difference** | **Meets Requirement?** |
| --- | --- | --- | --- | --- | --- | --- | --- | --- | --- | --- | --- |
|  |  | **1** | **2** | **3** | **4** | **5** | **6** | **7** | **8** |  |  |
| **1** | Random sequence | 7 | 7 | 7 | 4 | 7 | 7 | 8 | 3 | 4 | No |
|  | Allocation | A | A | A | B | A | A | B | A |  |  |
| **2** | Random sequence | 6 | 1 | 2 | 6 | 7 | 6 | 4 | 7 | 2 | Yes |
|  | Allocation | B | A | B | B | A | B | B | A |  |  |

**Step 3:** For subsequent permuted blocks, **block sizes of 8, 10, and 12** were randomly selected and varied. In these **equal blocks**, the number of participants allocated to **Group A and Group B was equal**. The order of block sizes was also determined using the online random number generator (<https://www.random.org/>). The first randomly selected equal block had a **block size of 8**.

**Step 4:** The researchers decided to **insert another unequal block after the fourth block**, repeating **Steps 1 and 2** to generate this new unequal block.

**Step 5:** After the insertion of the unequal block, permuted block randomisation resumed, again using **block sizes of 8, 10, and 12**. The first randomly selected block size after this insertion was **10**.

The mixed randomisation scheme used in this study is presented in Table 1.2.

Table 1.2 Mixed randomisation scheme for this study

| **Block Type** | Subject No. | Replacement randomisation sequence | Allocation | Group A Cumulative No. | Group B Cumulative No. |
| --- | --- | --- | --- | --- | --- |
| **Unequal block (n = 8, group size difference = 2)** | 1 | 6 | B | 0 | 1 |
|  | 2 | 1 | A | 1 | 1 |
|  | 3 | 2 | B | 1 | 2 |
|  | 4 | 6 | B | 1 | 3 |
|  | 5 | 7 | A | 2 | 3 |
|  | 6 | 6 | B | 2 | 4 |
|  | 7 | 4 | B | 2 | 5 |
|  | 8 | 7 | A | 3 | 5 |
|  |  |  |  |  |  |
| Equal block (n = 10) | 9 | 3 | A | 4 | 5 |
|  | 10 | 5 | A | 5 | 5 |
|  | 11 | 6 | B | 5 | 6 |
|  | 12 | 1 | A | 6 | 6 |
|  | 13 | 3 | A | 7 | 6 |
|  | 14 | 9 | A | 8 | 6 |
|  | 15 | 6 | B | 8 | 7 |
|  | 16 | 8 | B | 8 | 8 |
|  | 17 | 10 | B | 8 | 9 |
|  | 18 | 2 | B | 8 | 10 |
|  |  |  |  |  |  |
| Equal block (n = 12) | 19 | 2 | B | 8 | 11 |
|  | 20 | 7 | A | 9 | 11 |
|  | 21 | 4 | B | 9 | 12 |
|  | 22 | 8 | B | 9 | 13 |
|  | 23 | 6 | B | 9 | 14 |
|  | 24 | 3 | A | 10 | 14 |
|  | 25 | 9 | A | 11 | 14 |
|  | 26 | 10 | B | 11 | 15 |
|  | 27 | 5 | A | 12 | 15 |
|  | 28 | 12 | B | 12 | 16 |
|  | 29 | 11 | A | 13 | 16 |
|  | 30 | 7 | A | 14 | 16 |
|  |  |  |  |  |  |
| Equal block (n = 8) | 31 | 3 | A | 15 | 16 |
|  | 32 | 5 | A | 16 | 16 |
|  | 33 | 2 | B | 16 | 17 |
|  | 34 | 4 | B | 16 | 18 |
|  | 35 | 5 | A | 17 | 18 |
|  | 36 | 2 | B | 17 | 19 |
|  | 37 | 9 | A | 18 | 19 |
|  | 38 | 8 | B | 18 | 20 |
|  |  |  |  |  |  |
| **Unequal block (n = 8, group size difference = 2)** | 39 | 7 | A | 19 | 20 |
|  | 40 | 9 | A | 20 | 20 |
|  | 41 | 7 | A | 21 | 20 |
|  | 42 | 4 | B | 21 | 21 |
|  | 43 | 8 | B | 21 | 22 |
|  | 44 | 4 | B | 21 | 23 |
|  | 45 | 2 | B | 21 | 24 |
|  | 46 | 6 | B | 21 | 25 |
|  |  |  |  |  |  |
| Equal block (n = 10) | 47 | 3 | A | 22 | 25 |
|  | 48 | 9 | A | 23 | 25 |
|  | 49 | 4 | B | 23 | 26 |
|  | 50 | 7 | A | 24 | 26 |
|  | 51 | 8 | B | 24 | 27 |
|  | 52 | 7 | A | 25 | 27 |
|  | 53 | 5 | A | 26 | 27 |
|  | 54 | 6 | B | 26 | 28 |
|  | 55 | 2 | B | 26 | 29 |
|  | 56 | 10 | B | 26 | 30 |
|  |  |  |  |  |  |
| Equal block (n = 8) | 57 | 2 | B | 26 | 31 |
|  | 58 | 3 | A | 27 | 31 |
|  | 59 | 7 | A | 28 | 31 |
|  | 60 | 9 | A | 29 | 31 |
|  | 61 | 3 | A | 30 | 31 |
|  | 62 | 6 | B | 30 | 32 |
|  | 63 | 4 | B | 30 | 33 |
|  | 64 | 8 | B | 30 | 34 |
|  |  |  |  |  |  |
| Equal block (n = 10) | 65 | 9 | A | 31 | 34 |
|  | 66 | 5 | A | 32 | 34 |
|  | 67 | 3 | A | 33 | 34 |
|  | 68 | 7 | A | 34 | 34 |
|  | 69 | 6 | B | 34 | 35 |
|  | 70 | 8 | B | 34 | 36 |
|  | 71 | 5 | A | 35 | 36 |
|  | 72 | 10 | B | 35 | 37 |
|  | 73 | 2 | B | 35 | 38 |
|  | 74 | 4 | B | 35 | 39 |
|  |  |  |  |  |  |
| Equal block (n = 12) | 75 | 2 | B | 35 | 40 |
|  | 76 | 7 | A | 36 | 40 |
|  | 77 | 2 | B | 36 | 41 |
|  | 78 | 3 | A | 37 | 41 |
|  | 79 | 9 | A | 38 | 41 |
|  | 80 | 6 | B | 38 | 42 |
|  | 81 | 4 | B | 38 | 43 |
|  | 82 | 3 | A | 39 | 43 |
|  | 83 | 6 | B | 39 | 44 |
|  | 84 | 5 | A | 40 | 44 |
|  | 85 | 8 | B | 40 | 45 |
|  | 86 | 7 | A | 41 | 45 |

**Supplementary Material 2: Multi-Component Psychosocial Intervention Programme**

| Phases | | Support | Intervention indicators |
| --- | --- | --- | --- |
| Decision-making phase | Admission and Induce labour | Information support | Provide information leaflet and explain the principles, processes, and postoperative precautions of commonly induced labour methods for fetal malformations.  【When: the day of admission; How: face-to-face】 |
|  |  |  | Enumerate successful cases to enhance women's confidence in accepting induced labour.  【When: from admission to induction of labor; How: face-to-face】 |
|  |  | Social support | Invite women into a chatting group on WeChat where they can communicate with peers who have experienced termination of pregnancy for foetal anomaly.  【When: the first day of admission】 |
|  |  |  | Explain the care principles and precautions for family caregivers and encourage them to take good care of patients’ physical and mental health.  【When: the day of admission; How: Information leaflet.】 |
|  |  | Acceptance and Commitment Therapy | **Unit 1: A Brief Introduction to Acceptance and Commitment Therapy and Mindfulness Breathing**  1. Introduction to Acceptance and Commitment Therapy and Intervention content  2. Inviting women to openly express their feelings and experiences related to termination of pregnancy for foetal anomaly.  3. Guiding women to recognize their emotions and comprehend how these emotions influence them.  4. Experiencing mindful breathing with guided audio  5. Encouraging women to share their feelings and impressions regarding the mindful breathing exercise.  6. Intervention Summary and Assignment:  - Engage in mindfulness breathing exercises at least 1 or 2 times daily.  - Maintain a notebook to record the frequency and situations in which these exercises are practiced.  - Acceptance and Commitment Therapy and mindfulness-related articles were shared daily to support and encourage pregnant women in completing their daily assignments.  【When: the first day of admission; How: face-to-face, individual; Where: at a bedside in a curtained-off space or in a private room; Intervention duration: 40-60 min】 |
|  |  |  | **Unit 2: Creating Hopelessness, Defusion and Acceptance**  1. Recap: Talk with women about their mental health wellbeing and discuss their engagement in mindful breathing exercises since the last session.  2. Complete the *"Coping Strategy Worksheet"* and analyse the ineffective coping strategies currently employed by women, and how these ineffective coping strategies affect themselves and their families.  3. Tell the metaphors about how avoidance coping strategies would be exacerbating negative emotions:  a) "Red Elephant" Metaphor  b) "Quicksand" Metaphor  4. The "Mindfulness and Acceptance" meditation exercise to experience how to accept the negative emotions without avoidance.  5. The "Leaves on the stream meditation" exercise to experience the defusion with negative emotions.  6. Intervention Summary and Assignment:  - Advise women to do the "Drifting Leaves by the Stream" exercise when distressing thoughts arise.  【When: the second day of admission; How: face-to-face, individual; Where: at a bedside in a curtained-off space or in a private room; Intervention duration: 40-60 min】 |
|  |  |  | Supplementary metaphor: Self-Observation through Self-Scenery  1. Metaphor of "Sky and Weather"  2. Metaphor of "Chessboard"  By using these metaphors to help women observe their own inner emotions as they would a third person.  【When: The day before the induced abortion; How: face-to-face, individual; paper-based metaphors; Where: at a bedside in a curtained-off space or in a private room; Intervention duration: 20-30 min】 |
| Recovery phase | | Information support | Provide health education for women and their caregiver after induced labor, especially the treatment of postpartum breast distension.  【When: the day after induced labor; How: online via WeChat.】 |
|  |  |  | For women with future pregnancy needs, providing knowledge on prenatal care and considerations for subsequent pregnancies is essential. This includes factors contributing to fetal abnormalities, relevant assisted reproductive technologies, preconception and antenatal healthcare information, as well as fundamental knowledge on prenatal screening and diagnosis (Reference: "Prevention of Birth Defects" by Li Zhu, et al., Science Press). Additionally, offering online consultation links to genetic counseling experts' outpatient services can boost patients' confidence in pursuing subsequent pregnancies.  【When: throughout the recovery period, according to their needs; How: face-to-face】 |
|  |  | Social support | Facilitating peer experience sharing via Group Chat on WeChat to assist patients in adapting to the psychological changes resulting from the absence of the 'mother' role, thereby promoting their psychological recovery and self-esteem, enabling a swift transition into their appropriate social role.  【When: During hospitalization; How: online via WeChat. 】 |
|  |  |  | Encourage husbands and other family members to provide comfort and support; guide family members to identify signs of post-abortion depression and promptly seek professional psychological assistance when it arises; and guide family members to actively participate in caring for patients.  【When: during hospitalization; How: online via WeChat. 】 |
|  |  | Acceptance and Commitment Therapy | **Unit 3: Living in the Present - Mindfulness Practice**  1. Review: Care about women's current status, including thoughts, feelings, and emotions, and discuss the outcomes of previous exercises.  2. The metaphor of the "Time Machine" - Introducing the significance of living in the present moment.  3. "Eating Raisins" mindfulness exercise to experience the sensation of living in the present.  4. Intervention Summary and Assignment:  - Encourage the incorporation of mindful awareness practices into daily life, such as observing the texture and brushing technique while brushing teeth, paying attention to the height and length of each step while walking, noting whether the heel or the toe makes first contact, and being mindful of the movement of muscles and joints.  - Instruct patients to practice being fully present in everyday activities and to continue daily mindfulness breathing exercises.  【When: the first day after induction; How: face-to-face, individual; Where: at a bedside in a curtained-off space or in a private room; Intervention duration: 40-60 min】 |
|  |  |  | **Unit 4: Clarifying Values and Committing to Action**  1. Review: Care about women's current status, including thoughts, feelings, emotions, etc., and assess the progress of mindfulness practices. Ask pregnant women to rate their current life satisfaction on a scale of 0-10 and note the score they believe they can attain.  2. Identifying What Matters: Complete the *"Values Compass"* to assist patients in clarifying their values in different domains, i.e., "the life you want" and "what truly matters to you in life."  3. Set Goals and Commit to Actions: Fill out the *"Path to Growth"* to set achievable goals and commit to specific actions.  4. Intervention Summary and Assignment  - Reinforce practices from the six intervention sessions through the "Flexible Hexagon Exercise."  - Finally, express gratitude for the patient's participation, remind them that the skills learned during the intervention can be applied in their future, and recommend books about Acceptance and Commitment Therapy.  【When: the day of discharge; How: one-on-one offline; intervention location: at a bedside in a curtained-off space or in a private room; Intervention duration: 40-60min】 |

**Supplementary Material 3: Online Intervention Platform Based on WeChat.**


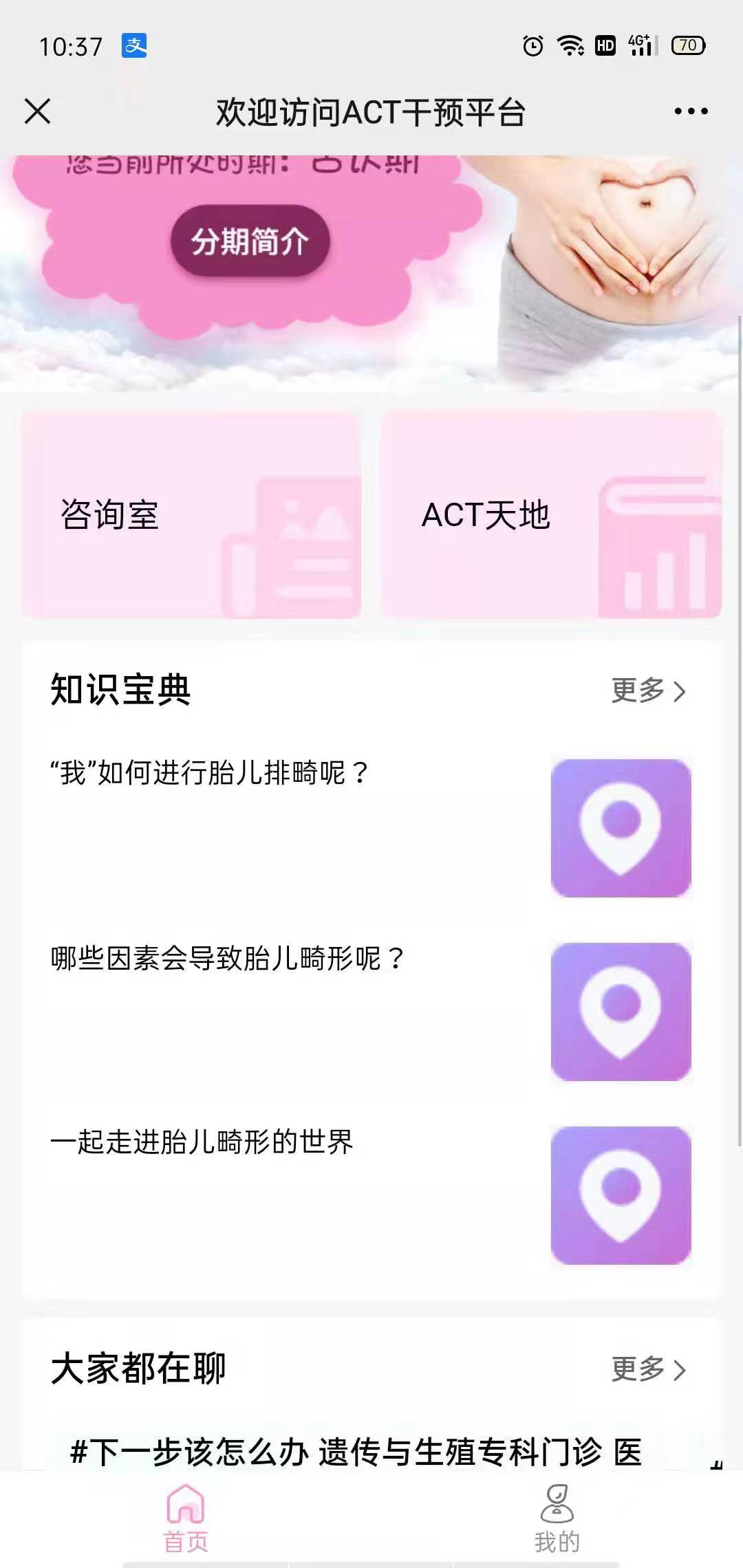

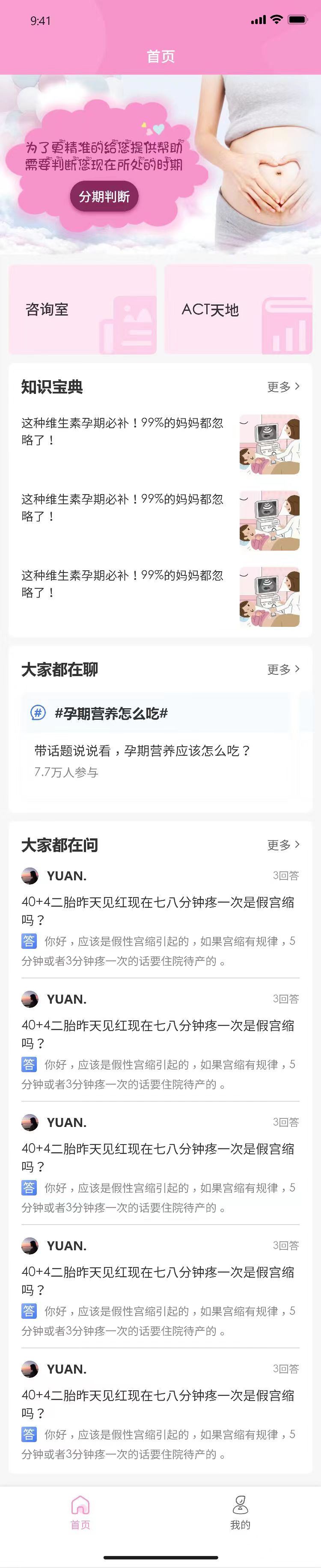


1. Guided by Schulz, K., Grimes, D.A., 2018. Essential Concepts in Clinical Research: Randomised Controlled Trials and Observational Epidemiology. Elsevier Health Sciences. [↑](#footnote-ref-2)
